# Erythropoietic indices in asthma patients on controller medications: a comparative analysis

**DOI:** 10.4314/ahs.v25i4.15

**Published:** 2025-12

**Authors:** Lawal Abdullahi, Lawal Rogo, Nura Garba, Danladi Bala, Hayatu Saidu

**Affiliations:** 1 Bayero University, Gwarzo road, PMB: 3011, Kano, Nigeria, University Health Services; 2 Bayero University, Gwarzo road, PMB: 3011, Kano, Nigeria, Department of Medical Laboratory Science; 3 Bayero University College of Health Sciences, Medical Laboratory Science

**Keywords:** Erythropoietic indices, asthma patients, comparative analysis

## Abstract

**Background:**

A key feature of asthma is hypoxia, which triggers erythropoietin (EPO) production to stimulate erythropoiesis and compensate for oxygen deficits. This study investigates the impact of asthma controller medications on erythropoietic response by evaluating serum EPO levels and reticulocyte counts among asthma patients at Murtala Muhammad Specialist Hospital, Kano.

**Methodology:**

This study is a comparative cross-sectional study involving 180 participants, comprising 60 asthmatics on controller medications, 60 treatment-naive patients with asthma, and 60 apparently healthy controls. Serum EPO levels were determined by ELISA, while complete blood count (CBC) and reticulocyte count were determined by automated hematology analyzer and manual method, respectively.

**Results:**

The study revealed significant differences in EPO levels (p = 0.00001), reticulocyte counts (p = 0.0004), and hematological parameters (p = 0.009) among the three groups. Treatment-naive asthmatics exhibited elevated reticulocyte counts (2.50 (IQR: 3.50)) and EPO levels (38.88 mIU/mL (IQR: 25.6 mIU/mL)), suggesting enhanced erythropoiesis in response to hypoxia. In contrast, asthmatics on controller medications showed a negative correlation (ϱ = - 0.564) between EPO levels and reticulocyte counts, indicating a potential suppressive effect of corticosteroids on erythropoiesis.

**Conclusion:**

Asthma is associated with increased erythropoietin production; it is however suppressed by inhaled corticosteroid therapy.

## Introduction

Asthma, a chronic respiratory condition affecting over 300 million people worldwide, significantly impacts daily life^1^. Characterized by chronic airway inflammation, airway hyperresponsiveness, and variable expiratory airflow limitation^2^. Different etiological factors including genetic, environmental factors are involved in development of asthma^3^. Among risk factors for asthma exacerbation: infection, allergen exposure, non-use of controller medication, non-white race and winter season^4^. Asthma triggers hypoxia, leading to increased erythropoietin (EPO) production and erythropoiesis^5^. In response to hypoxia, the body produces erythropoietin (EPO), stimulating erythropoiesis and increasing red blood cell production^5^. This compensatory mechanism involves the bone marrow producing more red blood cells, releasing erythrocytes into circulation to enhance oxygen delivery to tissues^6^. However, corticosteroids, commonly used to manage asthma, have multifaceted effects on erythropoiesis, influencing EPO production^7^, erythroid progenitor cell proliferation6, and red blood cell production^8^,^9^. While asthma's primary clinical features are well-documented, emerging evidence suggests potential systemic effects, including alterations in hematological parameters such as EPO and reticulocyte indices^8^-^11^. Limited data exist on how asthma influences these parameters, particularly in varying severities. In Nigeria, where asthma burden is rising and unique genetic and environmental factors influence disease outcomes^12^, local data is scarce, hindering efforts to contextualize findings and understand hematological implications among Nigerian patients. This study fills a critical knowledge gap by investigating the interplay between EPO, reticulocyte indices, and asthma in Nigeria.

## Methods

### Study design and settings

This study employed a comparative cross-sectional design to evaluate serum erythropoietin levels and reticulocyte indices among asthma patients on controller medication, controller medications -naive asthma patients (treatment naïve asthmatics), and apparently healthy non-asthmatic controls. Conducted at Murtala Muhammad Specialist Hospital in Kano, Nigeria.

### Participants and sample size determination

**Figure 1:**
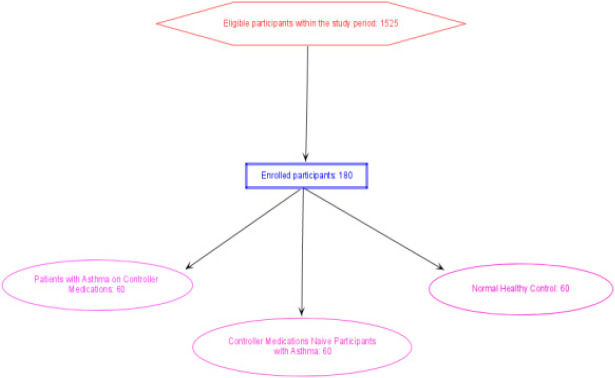
Recruitment of participants

The participants were patients with diagnosis of asthma receiving care at Murtala Muhammad Specialist Hopsital Kano. The study spanned from June to December, 2024. The study involved 180 adult participants comprising (60 asthmatics on controller medications, 60 controller medications -naive asthma patients, and 60 normal healthy controls (Figure 1). For the sample determination, we chose 95% confidence limit and minimum allowable error of 5% of the mean erythropoietin level. The participants were recruited from four locations/clinics of the hospital; the chest clinic, family clinic and emergency unit, the healthy controls were recruited from the donor clinic of the hospital. Random sampling (balloting) was used to recruit participants. Permission was obtained from the ethics committee of the ministry of health of Kano state. Informed consent was obtained from all the participants before recruitment. Exclusion criteria included respiratory comorbidities, recent blood transfusion, pregnancy, inflammatory diseases, severe kidney or liver disease.

### Data sources and measurements

For this research, the GINA criteria for the diagnosis of asthma in low and middle income countries was adopted: An individual said to have asthma when he/she has history of variable respiratory symptoms such as wheezing, coughing, shortness of breath and chest tightness; together with evidence of variable expiratory airflow limitations (peak airflow of <80% of the value expected for the patient's age and sex). Asthma patients on controller medications (inhaled corticosteroids) for at least six months constitute the test group. Asthma patients who were not on controller medication even if they have been managed with SABA only medication for acute attacks are considered controller medications naïve. Information on sociodemography, medical history of asthma, physical examination, lung function tests and medication history were collected using a standardized questionnaire. Blood samples were analyzed for serum erythropoietin levels using ELISA, complete blood count using an auto 3-part hematology analyzer, and reticulocyte count using manual microscopy. The study adhered to standard operating procedures and quality control measures to ensure accuracy and reliability of results. Data analysis was conducted using SPSS software (version 22.0). Due to non-normal distribution, data were summarized using median and interquartile range. Kruskal-Wallis H test and Spearman correlation analysis were used to compare and correlate variables, with statistical significance defined as p ≤ 0.05.

## Results

The socio-demographic characteristics of the 180 participants are summarized in Table 1. The sample included 60 asthmatic patients on controller medications, 60 controller medications -naive asthmatics, and 60 healthy controls. Females comprised 76.7%, 58.3% and 26.7% of the participants in asthmatics on controller medications, Controller Medications naïve and healthy controls respectively. Participants' ages ranged from 16 to 60 years, categorized into five groups: 16-20 years (15.0%), 21-30 years (31.1%), 31-40 years (25.0%), 41-50 years (18.3%), and 51-60 years (10.6%). Marital status varied among groups, with singles constituting 36.7% of asthmatics on medication, 35% of controller medications-naive asthmatics, and 73.3% of healthy controls, while married individuals made up 51.7%, 65%, and 26.7%, respectively. Occupational distribution showed that 48.3% were private workers, and 30.6% were without occupation. Nearly half (46.7%) of participants had attained higher formal education (Table 1).

**Table 1:** Socio-Demographic Characteristics of Study Participants

Socio-demographic Features	Asthmatics on controller Medications n(%)	Controller Medications naïve Asthmatics n(%)	Non Asthmatics n (%) Asthmatics	P-value
**Gender**				
Male	14 (23.3)	25 (41.7)	44 (73.3)	0.00001
Female	46 (76.7)	35 (58.3)	16 (26.7)	
Total	60 (100)	60 (100)	60 (100)	
**Age**(years)				
16-20	4 (6.7)	9 (15)	14 (23.3)	0.0001
21-30	13 (21.7)	14 (23.3)	29 (48.3)	
31-40	24 (40)	11 (18.3)	10 (16.7)	
41-50	18 (30)	9(15)	6 (10)	
51-60	1 (1.7)	17 (28.3)	1 (1.7)	
Total	60 (100)	60 (100)	60 (100)	
**Education Status**				
Tertiary	38 (63.3)	10 (16.7)	36 (60)	0.0023
Secondary	6 (10)	30 (50)	19 (31.7)	
Primary	8 (13.3)	6 (10)	4 (6.7)	
No formal western education education	8 (13.3)	14 (23.3)	1 (1.7)	
Total	60 (100)	60 (100)	60 (100)	
**Occupational status**				
No job including students	14 (23.3)	16 (26.7)	25 (41.7)	0.0002
Housewife	4 (6.7)	9 (15)	0 (0)	
Private worker	27 (45)	35 (58.3)	25 (41.7)	
Employer	15 (25)	0 (0)	10 (16.7)	
Total	60 (100)	60 (100)	60 (100)	
Marital Status				
Single	22 (36.7)	21(35)	44(73.3)	0.0001
Ssi				
Married	31(51.7)	39(65.0)	16(26.7)	
Divorced	4(6.7)	0	0	
Widow	3(5.0)	0	0	

Key: n= frequency, %=percentage.

The study participants displayed distinct behavioral patterns. Notably, all participants (100%) across the three groups—those with asthma on controller medications, controller medications -naive asthmatics, and non-asthmatics—were non-smokers and did not consume alcohol (Table 2). However, differences emerged in physical activity levels: 50% of asthmatics on medication, 70% of controller medications treatment-naive asthmatics, and 65% of non-asthmatics reported engaging in physical exercise, with a statistically significant difference observed (p = 0.064). Among exercisers, frequency varied, with 46.7% of asthmatics on medication, 54.8% of controller medications-naive asthmatics, and 23.1% of non-asthmatics exercising 1-2 times per week, showing a statistically significant difference, (p = 0.033) (Table 2).

**Table 2:** The Behavioral Characteristics of the Study Participants

Variables	Category	Asthmatics on controller Medications n(%)	Treatment naïve Asthmatics n(%)	Non Asthmatics n (%)	p-value
Cigarette Smoking	Non-smoker	60 (100)	60 (100)	60 (100)	
	Current smoker	0	0	0	
	Ex-smoker	0	0	0	
Alcohol Drinking Habit	YES	0	0	0	
	NO	60 (100)	60 (100)	60 (100)	
Physical Exercise	YES	30 (50)	42 (70)	39 (65)	0.064
	NO	30 (50)	18 (30)	21 (35)	
If yes, how often you do exercise	1-2 times/week	14 (46.7)	23(54.8)	9 (23.1)	0.033
	5. times/week	4 (13.4)	9 (21.4)	10 (25.6)	
	>5 times/week	12 (40)	10 (23.8)	20 (51.3)	

Key: n= frequency, %=percentage.

Table 3 highlights significant differences between asthmatics on controller medications (n=60) and controller medications -naive asthmatics (n=60) in several key areas. Asthmatics on controller medications were more likely to have a family history of asthma (50% vs 41.7%, p < 0.05) and a longer duration of asthma, with 86.6% having lived with the condition for over 15 months (p < 0.05).

**Table 3:** The Clinical Characteristics of the Asthmatic Patients

Variables	Category	Asthmatics on controller Medications n(%)	Controller Medications naïve Asthmatics n(%)	p-value
Family History	Yes	30 (50)	25 (41.7)	0.0015
	No	30 (50)	35 (58.3)	
Duration Patient live with asthma	<= 5months	4 (6.7)	9 (15)	0.005
	6-10 months	0	8 (13.3)	
	11-15 months	4 (6.7)	7 (11.7)	
	>15 months	52(86.6)	36 (60)	
Symptoms of asthma	Shortness of breath	39 (65.0)	52 (86.7)	0.0001
	Cough	26 (43.3)	48 (80.0)	0.002
	Wheezing	15 (25.0)	29 (48.3)	0.0021
	Tightness in the chest	17(28.3)	29 (48.3)	0.001
	Night time unrest	19(31.7)	27 (45.0)	0.133
Frequency of symptoms	1 time/week	36 ()	48 (80)	0.0001
	2-3 times/week	12 (20)	20 (33.3)	
	>3 times/week	0	1. (6.7)	

Key: n= frequency, %=percentage.

SABAs: short-acting beta 2 antagonists; ICS: inhaled corticosteroids; LTRAs: leukotriene receptor antagonist

**Figure 2:**
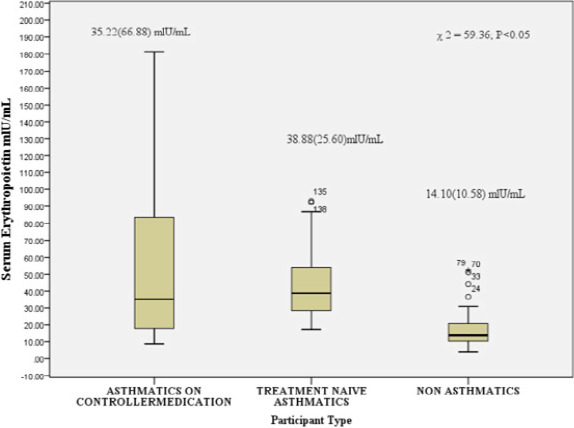
Comparison of Serum Erythropoietin Level Among the Groups. Kruskal- Wallis test was used to determine the significant difference

In contrast, controller medications -naive patients experienced more prevalent symptoms, including shortness of breath, cough, wheezing, and nighttime unrest (p < 0.05). Figure 2 presents a comparative analysis of erythropoietin (EPO) levels among three groups: asthmatic patients on controller medication, controller medications -naive asthmatic patients, and healthy controls. The median EPO levels were 35.22 mIU/mL (IQR: 66.8 mIU/mL) for asthmatics on medication, 38.88 mIU/mL (IQR: 25.6IU/mL) for controller medications -naive asthmatics, and 14.10 mIU/mL (IQR: 10.58 mIU/mL) for healthy controls. The Kruskal-Wallis test revealed a statistically significant difference among the groups, with a chi-square value of 59.63 (p < 0.05).

**Figure 3:**
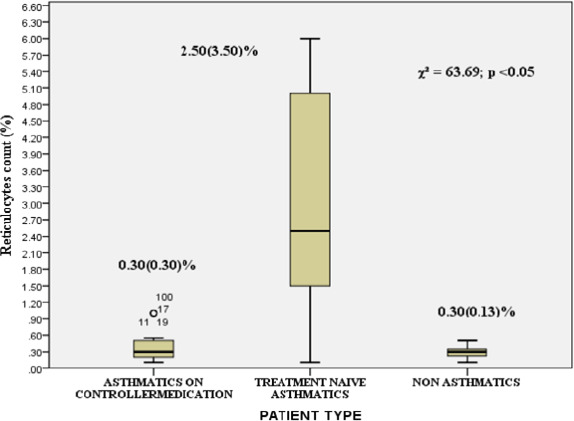
Comparison of Reticulocyte Count Among the Groups Kruskal- Wallis test was used to determine the significant difference

Figure 3 illustrates the median reticulocyte counts for the three groups: 0.30 (IQR: 0.30) for asthmatics on controller medication, 2.50 (IQR: 3.50) for treatment-naive asthmatics, and 0.30 (IQR: 0.13) for healthy controls. The Kruskal-Wallis test revealed a statistically significant difference among the groups (*χ*^2^ = 63.69, p < 0.05).

**Figure 4:**
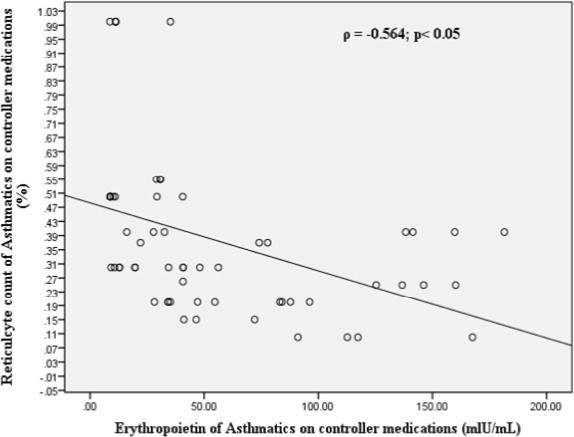
Spearman rank correlation between reticulocyte count and serum erythropoietin among the asthmatics on controller medication

Figure 4 shows a Spearman correlation analysis indicating a statistically significant negative correlation (ϲ = -0.564) between serum erythropoietin (EPO) levels and reticulocyte counts in asthmatic patients on controller medications. A comparative analysis of erythrocyte indices among asthmatics on controller medication (n=60), controller medications treatment-naive asthmatics (n=60), and healthy controls (n=60) revealed significant differences in several parameters (Table 4). The median values for RBC count were 4.74, 4.50, and 4.77 x 10^12^/L; hemoglobin (HGB) were 14.00, 13.60, and 13.85 g/dL; hematocrit (HCT) were 38.50, 36.90, and 39.30%; MCV were 81.70, 82.00, and 82.00 fL; MCHC were 35.75, 35.20, and 35.70 g/dL; MCH were 29.70, 28.90, and 28.25 pg; RDW-CV were 12.50, 13.40, and 13.80%; and RDW-SD were 41.00, 36.90, and 37.45 fL. Kruskal-Wallis tests showed significant differences among the groups for RBC count (*χ*^2^=9.42, p=0.009), HGB (*χ*^2^=9.16, p=0.010), HCT (*χ*^2^=6.87, p=0.032), RDW-CV (*χ*^2^=44.22, p<0.001), and RDW-SD (*χ*^2^=47.32, p<0.001).

## Discussion

Asthma, a chronic inflammatory disease of the airways, has been linked to alterations in erythropoiesis, the process by which red blood cells are produced^13^. Erythropoietin (EPO), a hormone produced by the kidneys, plays a crucial role in regulating erythropoiesis^14^. Our study investigated the effects of asthma on serum EPO levels and reticulocyte indices, revealing significant alterations in the hematological profile of asthmatic patients. The results of our study indicate that patients with asthma exhibit altered serum EPO levels, with mean serum EPO levels in asthmatic patients on controller medication and treatment-naïve asthmatics exceeding the normal range for human serum erythropoietin. These findings are consistent with previous research suggesting that chronic hypoxia, a hallmark of asthma, may lead to elevated serum EPO levels^5^,^15^,^16^. The elevated serum EPO levels in asthmatic patients may be an adaptive response to chronic hypoxia, aiming to enhance erythropoiesis and maintain oxygen delivery to tissues. However, our findings also highlight the complexity of the relationship between asthma, EPO, and erythropoiesis. For instance, the mean serum EPO levels in treatment-naïve asthmatics were higher than those in asthmatic patients on controller medication. This finding may be attributed to the inhibitory effects of cytokines on EPO production in the context of chronic inflammation^17^. A comparative analysis of reticulocyte indices among controller medications -naive asthmatics, asthmatics on controller medications, and normal healthy controls revealed significant differences in reticulocyte count (%). Controller medications -naive asthmatics exhibited substantially elevated reticulocyte counts (2.50 ± 3.50) compared to asthmatics on controller medications (0.30 ± 0.30) and normal healthy controls (0.30 ± 0.13). The elevated reticulocyte count in controller medications -naive asthmatics suggests increased erythropoiesis to compensate for chronic inflammation and oxidative stress associated with asthma^18^. However, despite the elevated EPO levels, the reticulocyte count was decreased or inappropriately normal among asthmatics on controller medications. This paradoxical finding may be attributed to the inhibitory role of glucocorticoids on erythroid differentiation and EPO signaling^19^,^20^. The steric mechanism underlying erythropoietin receptor inhibition by glucocorticoid receptor may involve physical interactions between the two receptors^20^. In the context of anemia associated with chronic inflammation, such as asthma, elevated levels of inflammatory cytokines may interfere with erythropoiesis, leading to decreased or inappropriately normal reticulocyte counts despite the presence of anemia^11^,^21^.

Our findings suggest that the use of controller medications in asthma may modulate erythropoiesis, leading to decreased reticulocyte counts despite elevated EPO levels. There is statistically significant negative correlation (r = -0.409) between serum EPO levels and reticulocyte counts. This negative correlation suggests that as serum EPO levels increase, reticulocyte counts decrease in asthmatic patients on controller medications. This finding appears paradoxical, as EPO is expected to stimulate erythropoiesis, leading to increased reticulocyte production^22^. However, several factors may contribute to this observed negative correlation. One possible explanation is the inhibitory effect of glucocorticoids, which are commonly used in controller medications for asthma, on erythroid differentiation and EPO signaling^19^,^20^. Glucocorticoids may suppress the erythropoietic response to EPO, leading to decreased reticulocyte production despite elevated EPO levels. Another possible explanation is the presence of chronic inflammation in asthma, which can lead to anemia of chronic disease^21^. In this context, elevated EPO levels may not be sufficient to overcome the inhibitory effects of inflammatory cytokines on erythropoiesis, resulting in decreased reticulocyte production. The negative correlation between serum EPO levels and reticulocyte counts in asthmatic patients on controller medications underscores the complexity of the relationship between EPO and erythropoiesis in this population. This paradoxical finding suggests that multiple factors, including glucocorticoid therapy and chronic inflammation, may modulate the erythropoietic response to EPO, leading to a disconnect between EPO levels and reticulocyte production. The hematological profiles of asthmatic patients were significantly different from the healthy controls, regardless of controller medications use, as evident from the results presented in Table 4. Specifically, treatment-naïve asthmatics exhibited higher red blood cell (RBC) counts compared to asthmatic patients on controller medication and healthy controls. This finding suggests an adaptive response to chronic hypoxia associated with asthma. Previous studies have reported conflicting findings regarding the effects of asthma on hematological parameters. For instance, Saidu9 found reduced RBC counts, packed cell volume, hemoglobin concentration, and red cell indices in asthmatics on inhaled corticosteroids. In contrast, EjazlO reported elevated hemoglobin concentration and hematocrit in a group of Pakistani asthmatics. The absence of significant differences in mean corpuscular volume (MCV), mean corpuscular hemoglobin concentration (MCHC), and mean corpuscular hemoglobin (MCH) among the three groups suggests that RBC size and hemoglobinization are relatively preserved. However, significant variations in red cell distribution width-coefficient of variation (RDW-CV) and red cell distribution width-standard deviation (RDW-SD) among the groups indicate differences in RBC size distribution. The effects of corticosteroids on erythropoiesis are complex and multifaceted. Corticosteroids have been shown to affect the maturation of erythroid cells through transcriptional mechanisms and by direct effects on erythropoietin signaling^20^. However, the magnitude of this effect is not clinically significant, and may be influenced by the route of administration^23^. The higher RBC counts in treatment-naïve asthmatics may be attributed to the increased EPO production in response to chronic hypoxia, leading to enhanced erythropoiesis. However, the decreased reticulocyte counts in asthmatic patients on controller medication, despite elevated EPO levels, may be due to the inhibitory effects of glucocorticoids on erythroid differentiation and EPO signaling. The significant variations in RDW-CV and RDW-SD among the groups may indicate differences in RBC size distribution, which could be influenced by factors such as inflammation, oxidative stress, and erythropoietic activity. Further studies are warranted to investigate the underlying mechanisms and clinical implications of these findings.

**Table 4:** Comparison of Erythrocyte Profile Pattern Among Asthmatics on Controller Medication, Treatment Naïve Asthmatics, and Normal Healthy Control Participants

Variables	Control (n=60)	Asthmatics on controller medication (n=60)	Controller Medications naïve asthmatics (n=60)	*χ*^2^-statistic	p-value
RBC count (X10^12^/L)	4.74(0.24)	4.50(0.57)	4.77(0.45)	9.42	0.009
HGB (g/dL)	14.00(0.70)	13.60(1.70)	13.85(1.10)	9.16	0.010
HCT (%)	38.50(1.85)	36.90 (4.68)	39.30(6.60)	6.87	0.032
MCV (fL)	81.70(2.90)	82.00 (11.35)	82.00(7.20)	1.15	0.564
MCHC (g/dL)	35.75(2.38)	35.20 (1.60)	35.70(2.98)	0.771	0.68
MCH (pg)	29.70(2.32)	28.90(1.80)	28.25(3.30)	4.63	0.099
RDW-CV (%)	12.50(0.93)	13.40(1.20)	13.80(1.40)	44.22	0.0001
RDW-SD (fL)	41.00(2.25)	36.90(4.82).	37.45(3.60)	47.32	0.0001

The differences in the parameters between three groups were compared (using Kruskal-Wallis test). n= group sample size. Values are expressed as Median (IQR).

Key: RBC, red blood cell, HGB, hemoglobin; MCV: mean cell volume, HCT: Haematocrit, MCH, mean cell hemoglobin; MCHC: mean cell hemoglobin concentration, RDW-SD: red cell distribution width-standard deviation RDW-CV: red cell distribution width-coefficient of variation.

## Conclusion

Controller medication naïve asthmaticshave higher serum EPO levels and reticulocyte counts compared to controller medication experienced asthmatics and normal healthy individuals.
